# Diagnosing Impaired Glucose Tolerance Using Direct Infusion Mass Spectrometry of Blood Plasma

**DOI:** 10.1371/journal.pone.0105343

**Published:** 2014-09-09

**Authors:** Petr G. Lokhov, Oxana P. Trifonova, Dmitry L. Maslov, Elena E. Balashova, Alexander I. Archakov, Ekaterina A. Shestakova, Marina V. Shestakova, Ivan I. Dedov

**Affiliations:** 1 Institute of Biomedical Chemistry RAMS, Moscow, Russia; 2 Endocrinological Scientific Centre, Moscow, Russia; Aligarh Muslim University, India

## Abstract

The goal of this study was to evaluate the capacity for mass spectrometry of blood plasma to diagnose impaired glucose tolerance (IGT). For this study, blood plasma samples from control subjects (n = 30) and patients with IGT (n = 20) were treated with methanol and low molecular weight fraction were then analyzed by direct infusion mass spectrometry. A total of 51 metabolite ions strongly associated with IGT were detected. The area under a receiver operating characteristic (ROC) curve (AUC) for diagnosing IGT that was based on an analysis of all these metabolites was 0.93 (accuracy 90%, specificity 90%, and sensitivity 90%). The associated reproducibility was 85%. The metabolites identified were also consistent with risk factors previously associated with the development of diabetes. Thus, direct infusion mass spectrometry of blood plasma metabolites represents a rapid, single-step, and reproducible method for the analysis of metabolites. Moreover, this method has the potential to serve as a prototype for clinical analyses that could replace the currently used glucose tolerance test with a more patient-friendly assay.

## Introduction

Impaired glucose tolerance (IGT) is a pre-diabetic state that is associated with insulin resistance and an increased risk of cardiovascular pathology. Moreover, IGT has been shown to precede type 2 diabetes mellitus by many years [1]. To prevent or delay the development of diabetes in pre-diabetic individuals, changes in diet and increased physical activity are recommended [2], [3]. Currently, the oral glucose tolerance test (OGTT) represents the ‘gold standard’ for detecting IGT. However, this test has exhibited low reproducibility despite being considered useful for a diagnosis of IGT, as well as for diabetes and other cardiovascular risk factors [4]–[6]. In addition, an OGTT is time consuming (takes 2 h) and some people may experience sugar shock during it [7]. Therefore, a more rapid and reproducible test for diagnosing IGT is needed.

In this study, a metabolomics approach was evaluated for its ability to diagnose IGT. In metabolomics, a large number of small molecules (metabolites) can be detected in samples, and in the case of bodily fluid samples, this capacity provides great potential for the development of diagnostic assays [8], [9]. Previously, the majority of metabolomic studies of blood plasma samples have been conducted using multi-stage protocols [10], and numerous diagnostic metabolites have been identified, including metabolites related to a pre-diabetic state. More recently, prospective nested case control studies identified five branched chain and aromatic amino acids as predictors of type 2 diabetes [11]. In another study, the metabolites, glycine, lysophosphatidylcholine, and acetylcarnitine, exhibited significantly altered levels in patients with IGT compared to individuals with normal glucose tolerance [12].

Of the available metabolomics technologies, direct infusion mass spectrometry appears to be the most suitable for clinical application. Using this technique, biological materials can be directly applied to an ionization source of a mass spectrometer without any preliminary separation, and the capacity for this approach to be used for diagnostics has been demonstrated in previous studies [13]–[17]. Consequently, direct infusion mass spectrometry can characterize a metabolome without additional distortion being introduced by a separation step. Moreover, this should simplify the translation of this metabolomics-based method into the clinic. Therefore, in the present study, direct infusion mass spectrometry (DIMS) of blood plasma metabolites was performed to evaluate this method as a diagnostic assay for a pre-diabetic state characterized by IGT.

## Materials and Methods

### Patient cohorts

Study participants were recruited at the Polyclinic Department of the Endocrinology Research Centre (Moscow, Russia). The study was approved by the ethical review committee #27-01 of the RAMS (Moscow, Russia), approval number #64 (statement # 01-02/62). Subjects at risk for diabetes who were admitted to the department were selected to participate in this study. All participants signed their written informed consent to provide blood samples for research purposes. Blood plasma concentrations of diagnostic substances (glucose, uric acid, total cholesterol, insulin, triglycerides, low-density lipoprotein (LDL), and high-density lipoprotein (HDL) were measured using the Architect c4000 clinical chemistry analyzer (Abbott Diagnostics, Abbott Park, IL, USA). Glycated hemoglobin (HbA1c) was measured using the Bio-Rad D10 hemoglobin testing system (Bio-Rad Laboratories, France). For the oral glucose talerance test (OGTT), a standard glucose dose (75 g) was orally ingested and blood glucose levels were checked two hours later. IGT was diagnosed if the post-load glucose levels were between 7.8 and 11.0 mmol/l (W.H.O. 1999) [18]. In this study, OGTT results were used to establish gender-matched cases (IGT; n = 20) and control (Normal; n = 30) groups. Table 1 (and Table S1) presents the clinical characteristics of the cohort.

**Table 1:** Clinical characteristics of patient cohort.

CHARACTERISTICS	VALUES (average ± SD/range)		AUC	t-test (p-value)
	Control subjects	Subjects with IGT		
**Number**	**30**	**20**	**-**	**-**
**Sex (male/female)**	**15/15**	**10/10**	**0.51**	**-**
**Age (years)**	**53.3±14.0/32–82**	**61.8±12.2/38–85**	**0.66**	**0.03**
**BMI (kg/m^2^)**	**35.0±8.2/24.5–53.2**	**33.9±8.9/23.2–57.0**	**0.46**	**0.66**
**Fasting glucose (mmol/l)**	**5.5±0.4/4.8–6.4**	**5.6±0.4/5–6.1**	**0.55**	**0.51**
**Glucose in OGTT (mmol/l)**	**6.4±1.0/4.1–7.8**	**9.8±1.0/8.2–11.0**	**1.00^a^**	**0.00**
**Insulin (µU/ml)**	**16.1±20.0/3.3–100.8**	**14.5±7.9/4.9–31.7**	**0.59**	**0.74**
**HbA1c (%) [mmol/mol]**	**5.8±0.4/5.1–6.4 [40±4.4/32–46]**	**6.1±0.4/5.4–6.6 [43±4.4/36–49]**	**0.77**	**0.001**
**LDL (mmol/l)**	**3.4±0.7/1.7–5.3**	**3.1±1.0/1.6–4.7**	**0.41**	**0.16**
**HDL (mmol/l)**	**1.2±0.4/0.7–1.9**	**1.1±0.4/0.6–1.9**	**0.40**	**0.23**
**Cholesterol (mmol/l)**	**5.2±0.8/3.9–6.6**	**5.1±1.2/3.0–6.8**	**0.47**	**0.58**
**Uric acid (µmol/l)**	**374±82/223–514**	**386±83/265–581**	**0.54**	**0.61**
**Triglycerides (mmol/l)**	**1.3±0.6/0.5–3.0**	**1.69±0.9/0.8–3.9**	**0.62**	**0.07**
**HOMA-IR**	**4.0±4.8/0.8–24.2**	**3.6±2.1/1.1–8.6**	**0.59**	**0.79**
**HOMA-β**	**160±202/31–1061**	**139±76/55–283**	**0.58**	**0.65**
**_mb_GTT**	**9.9±7.2/0–26**	**32.3±9.9/10–46**	**0.93**	**0.0000**

a) The AUC for glucose (OGTT) is equal to 1 since the OGTT test was used to establish control and IGT groups.

*AUC*, a receiver operating characteristic (ROC) curve; *OGTT*, oral glucose tolerance test; *_mb_GTT*, mass spectrometry-based GTT; *HOMA*, homeostatic model assessment; *BMI*, body mass index.

### Blood sampling and sample preparation

Blood samples for metabolomic analysis were taken from the vein before the morning meal. Samples (3 ml) were placed into glass tubes containing K_2_EDTA (BD Vacutainer; Becton, Dickinson and Company, Franklin Lakes, NJ, USA) and centrifuged within 15 min of blood collection at 1600×g and room temperature. The resultant blood plasma was subdivided into aliquots that were pipetted into plastic tubes. These tubes were marked, transported in special thermo containers, frozen, then stored at −80°C until analysis. The analyzed samples were subjected to one freeze/thaw cycle. To test the reproducibility of this protocol, an additional set of blood samples (n = 20) were collected from the same individuals within 2–7 days of the original collection.

For plasma deproteinization, aliquots (10 µl) were mixed with 10 µl water (LiChrosolv; Merck KGaA, Darmstadt, Germany) and 80 µl methanol (Fluka, Munich, Germany) and incubated at room temperature. After 15 min, samples were centrifuged at 13000×g (MiniSpin plus centrifuge; Eppendorf AG, Hamburg, Germany) for 10 min. Deproteinized supernatants were then transferred to clean plastic Eppendorf tubes, and fifty volumes of methanol containing 0.1% formic acid (Fluka) was added to each tube. The resulting solutions were subjected to mass spectrometry analysis.

### Mass spectrometry analysis

Samples were analyzed with a maXis hybrid quadrupole time-of-flight mass spectrometer (Bruker Daltonics, Billerica, MA, USA) equipped with an electrospray ionization (ESI) source (Supporting Information S1). The mass spectrometer was set up to prioritize the detection of ions with a mass-to-charge ratio (m/z) ranging from 50 to 1000, with a mass accuracy of 1–3 parts per million (ppm). Spectra were recorded in the positive ion charge detection mode. Samples were injected into the ESI source using a glass syringe (Hamilton Bonaduz AG, Bonaduz, Switzerland) connected to a syringe injection pump (KD Scientific, Holliston, MA, USA). The flow rate of samples to the ionization source was 180 µl/h, and samples were injected in a randomized order (e.g., control samples were run between case samples). Mass spectra were obtained using DataAnalysis version 3.4 (Bruker Daltonics) to summarize one minute signals. Ion metabolite masses were determined from the mass spectrum peaks obtained using the DataAnalysis program. All peaks above noise level (singal to noise ration >1) were selected, and the metabolite ion masses were pooled and processed using Matlab version R2010a (MathWorks, Natick, MA, USA). Alignment of mass peaks was performed as described previously [16]. This and all other calculations were performed using Matlab software.

For mass spectrometric peaks included in IGT pattern and having clear isotope patterns, the correspondence to the specific metabolites from the database “Human Metabolome Database” (http://www.hmdb.ca) [19] and/or Metlin (Scripps Center for Mass Spectrometry, USA; http://metlin.scripps.edu) [20] was established. Theoretical isotope patterns for each of these metabolites were generated using the Molecular Weight Calculator v.6.46 program (http://ncrr.pnl.gov).

### Score calculation for the mass spectrometry-based GTT

Metabolite ions with peak intensities strongly associated with IGT (n = 51) were included in the calculation of a mass spectrometry-based glucose tolerance test (_mb_GTT) score. To this end, the intensity of each peak was considered as a measure for a separate two-state test, where the final _mb_GTT score was represented as the number of positive results from these tests. To define the threshold values that would separate positive and negative results for all single-ion tests and the final mbGTT score, ROC curves were generated using the *rocplot* function of the Matlab program. This function returns all required thresholds with the corresponding accuracies, sensitivities, specificities, and area under ROC curve (AUC) values. Mass peaks having intensities near the noise level observed for more than 10% of the samples (i.e., corresponding metabolites' concentrations were near the limit-of-detection (LOD), and therefore, potentially did not exhibit valid distributions in the mass spectral data) were not included in calculations of _mb_GTT scores.

### Metabolic pattern of IGT

A metabolic pattern was established using a list of ion masses strictly associated with IGT and which were detected using ESI-DIMS. For each metabolite ion, the threshold for the mass peak intensity (derived from the metabolite concentration) was defined in order to separate positive and negative results for IGT, and these threshold values were expressed in quintiles that were defined based on the control set of mass spectra (n = 30). For example, if the intensity of a mass peak with a m/z of 133.097 is higher than that of 0.57 quintiles, i.e., 133.097^0.57^, then the _mb_GTT score should be increased by one. For metabolite ions expressing a lower intensity with IGT (these ion masses are underlined in the pattern), the _mb_GTT score should be increased if the intensity of a mass peak lower than that is defined by the quintile.

The _mb_GTT scores were additionally validated using the *leave-one-out* method [21]. This method involves the one-by-one removal of each data point (sample) from the dataset and determination of the resulting _mb_GTT score based on the remaining data. This _mb_GTT score was then tested by reintroducing the sample which was not included. Thus, _mb_GTT scores were obtained and tested for all 50 samples.

## Results

### Direct infusion mass spectrometry identifies a diagnostic pattern of IGT

**Figure 1:**
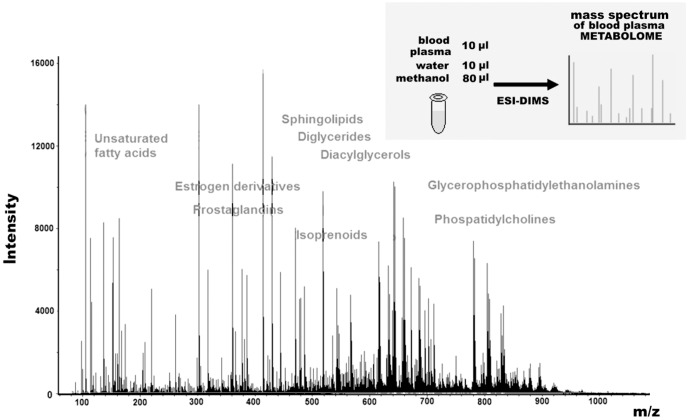
A typical mass spectrum of human plasma metabolites. This mass spectrum was obtained following the direct injection of a deproteinized blood plasma sample into an electrospray ion source of a hybrid quadrupole time-of-flight mass spectrometer. The main metabolite groups detected are labeled. The embedded figure in the right upper corner shows the single-stage workflow that was used to obtain the corresponding metabolome profile of blood plasma by ESI-DIMS.

ESI-DIMS analysis of plasma samples resulted in the detection of ∼4000 low weight molecular ions per sample (Fig. 1). Based on the peak intensity values obtained, AUC values were calculated for each metabolite. Fifty-one ions were strongly associated with IGT were subsequently selected to design a diagnostic pattern for IGT. Of these, 35 ions exhibited an increase in concentration with IGT (with an AUC value >0.7), and 16 ions exhibited a decrease in concentration with IGT (with an AUC value >0.76): 133.097^0.57^; 135.039^0.68^; 149.057^0.72^; 165.089^0.71^; 175.145^0.71^; 177.124^0.89^; 178.993^0.85^; 181.084^0.85^; 200.973^0.75^; 204.952^0.77^; 211.004^0.64^; 223.094^0.80^; 228.924^0.72^; 239.014^0.81^; 248.242^0.78^; 250.045^0.71^; 256.155^0.79^; 256.261^0.81^; 263.085^0.88^; 272.943^0.70^; 278.244^0.79^; 280.264^0.92^; 282.279^0.78^; 284.295^0.89^; 296.221^0.78^; 297.228^0.63^; 302.245^0.82^; 310.874^0.80^; 312.327^0.66^; 324.253^0.61^; 366.833^0.77^; 367.119^0.90^; 376.810^0.75^; 434.819^0.82^; 494.773^0.92^; ***114.896^0.23^; 116.896^0.16^; 122.925^0.23^; 124.923^0.23^; 139.914^0.16^; 152.046^0.43^; 172.854^0.19^; 238.839^0.29^; 248.868^0.16^; 249.872^0.09^; 250.865^0.33^; 256.981^0.19^; 260.896^0.19^; 298.795^0.23^; 304.740^0.19^; 306.827^0.16^***.

**Figure 2:**
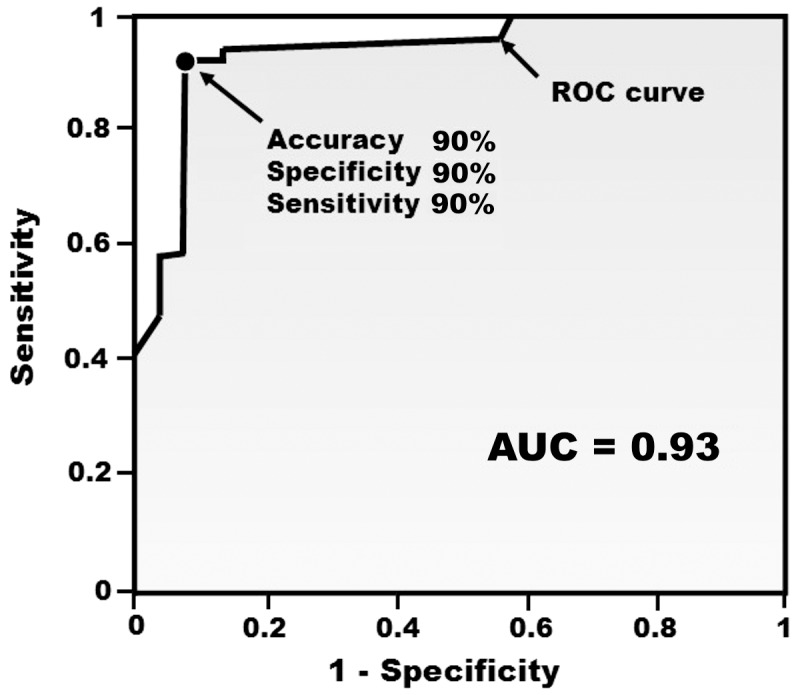
ROC curve for the _mb_GTT. A total of 50 samples from IGT cases (n = 20) and control individuals (n = 30) were used to build a ROC curve. The _mb_GTT score was based on data for 51 metabolite ions. The point represented on the ROC curve represents the maximum _mb_GTT accuracy value. The area under the ROC curve (AUC) is shaded.

The AUC value for the _mb_GTT based on this pattern was 0.93. A _mb_GTT score of 22 units was identified as the threshold value for distinguishing IGT versus normal states, and this score was associated with a maximum accuracy value of 90%, a sensitivity value of 90%, and a specificity value of 90% (Fig. 2). The reproducibility of the _mb_GTT score that was calculated for 20 subjects was 85%. In addition, the accuracy, sensitivity, and specificity for this _mb_GTT score were measured using the *leave-one-out* test, and the values obtained were 88%, 90%, and 87%, respectively.

### Metabolite identification

Metabolites associated with IGT that were identified from the samples analyzed based on high AUC values included fatty acids, amides of fatty acids, and five other metabolites: butanediol, phosphoglycolic acid, p-cresol sulfate, ornithine, and phosphatidylcholine (Table 2). In addition, metabolites present at lower concentrations, yet having high AUC values, were found to represent quasi-molecular ions containing potassium ions.

**Table 2:** Blood plasma metabolites with high AUC values that are associated with IGT.

#	Metabolite	Identification # in database^a^	Molecular weight		Detected Ion	Elemental composition	Wilcoxon test (p-value)	AUC
			Measured (m/z)	Calculated (Da)				
								
**Increased with IGT**								
1	2,3-Butanediol	HMDB03156	135.0390	135.0392	[M+2Na-H]^+^	C_4_H_10_O_2_	0.0027	0.76
2	Linoleamide	Metlin ID 43435	280.2631 302.2432	280.2635 302.2454	[M+H]^+^ [M+Na]^+^	C_18_H_33_NO	0.0012 0.0101	0.77 0.71
3	Oleamide	HMDB02117	282.2778	282.2791	[M+H]^+^	C_18_H_35_NO	0.0178	0.71
4	Stearamide	HMDB34146	284.2916	284.2948	[M+H]^+^	C_18_H_37_NO	0.0052	0.74
5	Decenedioic acid	HMDB00603	223.0943	223.0941	[M+Na]^+^	C_10_H_16_O_4_	0.0288	0.72
6	α- (or β-)ketooctanoic acid	HMDB13211 (or HMDB10721)	181.0827	181.0835	[M+Na]^+^	C_8_H_14_O_3_	0.0335	0.71
7	Octenoic acid	HMDB00392	165.0859	165.0886	[M+Na]^+^	C_8_H_14_O_2_	0.0183	0.73
8	Malic acid	HMDB00156	178.9882	178.9927	[M+2Na-H]^+^	C_4_H_6_O_5_	0.0204	0.72
9	Glucuronic acid	HMDB00127	239.0164	239.0138	[M+2Na-H]^+^	C_6_H_10_O_7_	0.0303	0.71
10	Phosphoglycolic acid	HMDB00816	200.9532 156.9831	200.9535 156.9897	[M+2Na-H]^+^ [M+H]^+^	C_2_H_5_O_6_P	0.016	0.74
11	p-Cresol sulfate	HMDB11635	211.0033	211.0035	[M+Na]^+^	C_7_H_8_O_4_S	0.0050	0.76
12	Ornithine	HMDB00214	133.0962	133.0972	[M+H]^+^	C_5_H_12_N_2_O_2_	0.0227	0.72
13	Phosphatidylcholine	HMDB08097	366.7848	366.7805	[M+2H]^2+^	C_40_H_78_NO_8_P	0.058	0.71
**Decreased with IGT**								
14	K_2_Cl^+^	-	112.8943	112.8957	-	-	0.0027	0.79
15	K_2_NaCl_2_^+^	-	170.8530	170.8543	-	-	0.0335	0.79

a) ‘Metlin ID’ refers to an identification number in the METLIN metabolite database, and ‘HMD’ refers to an identification number in the Human Metabolome Database. *AUC*, a receiver operating characteristic (ROC) curve; *IGT*, impaired glucose tolerance.

## Discussion

### Development of an _mb_GTT

Mass spectrometry techniques that are currently available facilitate the capture of high-throughput ‘snapshots’ of the metabolome [8], [14]. Moreover, for the analysis of bodily fluids, mass spectrometry has exhibited great potential for its application in diagnostic assays. In the present study, DIMS of plasma samples using an ESI source in positive ion mode (optimal for the ionization of many blood plasma substances [22]), resulted in the detection of ∼4000 metabolite ions per sample. This comprehensive dataset provided valuable insight into the metabolome of blood plasma, and also demonstrated the potential for this approach to diagnose metabolic disorders related to prediabetes.

### Efficacy of the _mb_GTT

Calculation of an AUC value is an ideal method for classifying the efficacy of a two-state test. For example, it was previously demonstrated that AUC values ranging from 0.5–0.6 indicate a test does not work; 0.6–0.7, a poor, yet functional, test; 0.7–0.8 – a good test, and 0.9–1.0 – an excellent test [23]. The AUC value for the _mb_GTT performed in the present study was 0.93, thereby classifying this test as having excellent efficacy. However, the _mb_GTT did not achieve maximum accuracy based on the use of OGTT results to distinguish IGT and control groups.

An AUC value comprehensively characterizes the diagnostic power of the two-state test, to which the _mb_GTT also belongs, and threshold values specified in the IGT pattern are defined using the control set of samples (i.e., the samples influence the diagnostic pattern). Correspondingly, the capacity for the _mb_GTT to diagnose IGT was additionally validated using the *leave-one-out* test. Negligible decreases that were observed in the diagnostic parameters of the *leave-one-out* test relate to slight fluctuations in the threshold values from one run to another during testing. More stable threshold values could be established if a larger control set was used to define the quintiles.

### Reproducibility of the _mb_GTT

The low reproducibility of the OGTT is a key shortcoming of this assay [4]–[6]. Moreover, glucose levels, as well as the level of most metabolites in blood, can widely vary. For example, the coefficient of variation (CV) for glucose levels for a 2 h OGTT is ∼25% [4], thereby leading to very low reproducibility of the OGTT test for diagnosing IGT. Typically, such a CV value is not acceptable for bioanalytical assays where a CV of 15% is considered a maximum permissible value [24].

Metabolite concentrations measured by mass spectrometry also generally exhibit a very high CV (median CV of 46%) [25]. As a result, mass spectrometry-based metabolomic tests are characterized by very low reproducibility, and this prevents their implementation in clinics [26]. However, the _mb_GTT overcomes this shortcoming by using a score calculation method which averages metabolite fluctuations. Thus, the _mb_GTT can accommodate the concomitant increase and decrease in levels of different subsets of metabolites. Ideally, when metabolite levels are independent from each other and their number is high, the CV of _mb_GTT scores should approach zero. The CV for the _mb_GTT score obtained in the present study was 8.4%, which is significantly better than the CV value permissible for bioanalytical assays.

### Mass spectrometry pattern of IGT

An IGT pattern was identified from metabolite ion masses strictly associated with the disease state. For each metabolite ion, a threshold value was established for the mass peak intensity in order to distinguish positive and negative results for the IGT state. The mass values presented in the pattern are expressed in absolute units (i.e., m/z, which is generally equal to daltons). Mass peak intensities, derived from each metabolite's concentration in blood, are expressed in units which depend on the type, model, and settings of the mass spectrometer, as well as detector consumption, purity of used solutions, the operating state of the ion source and ion transferring system, and the exact pH value of the samples. Therefore, these units are not reproducible from one mass spectrometer to another. To overcome this problem and to make the IGT pattern acceptable for diagnostics, defined threshold values were expressed in quintiles.

Generally, if one variable is higher than another, this will be detected by an instrument. If the variables have a range according to their values, this order should be preserved independently from the instrument used (i.e., the order of variables cannot be changed). This statement is the basis for establishing a metabolic pattern for IGT that can be adapted for different mass spectrometers. Quintiles are often used to set cut-off points for a given dataset. For example, a 0.3 quintile defines a threshold that separates 30% of the lower variables from the others, and this set of variables, as well as other sets separated by quintiles, are non-alterable and independent from the measuring instrument used. Therefore, the IGT pattern with threshold values expressed in quintiles represents an acceptable method for accommodating the use of different mass spectrometers.

### Metabolite contributions to the _mb_GTT

The identification of the metabolites which contributed to the _mb_GTT results was an additional step that was performed to validate the proposed approach for diagnosing IGT. Blood plasma signatures for prediabetes have been well-characterized, including recent data from metabolomics studies [11], [12]. In particular, amides of the fatty acids, also known as endocannabinoids, play an important role. For example, activation of the endocannabinoid system has been shown to increase food intake, promote weight gain [27], [28], and can also contribute to the worsening of a cardiovascular profile (i.e., body weight, body mass index (BMI), waist circumference, insulin and adiponectin levels) [29]. The increased endocannabinoid levels detected in IGT patients in the present study is consistent with these data. Increased fatty acid levels were also detected in subjects with IGT, and this is consistent with previously published data as well. It is known that prediabetic metabolic syndrome has been characterized by increased levels of lipids [30], including fatty acids [31].

Decenedioic acid is a dicarboxylic acid. For diabetic patients, increased urinary excretion of dicarboxylic acids occurs, and is considered to be a marker of an oxidative attack on fatty acids [32]. Increased levels of decenedioic acid were detected in the present study, and may represent evidence that this oxidative attack can occur in a prediabetic state. However, further studies will be needed to confirm this.

**Phosphatidylcholine with the elemental composition** C_40_H_78_NO_8_P was present at higher levels in the IGT patients analyzed in this study. Phosphatidylcholine may also be related to metabolic syndromes associated with IGT that are characterized by lipid disorders.

α-κetooctanoic acid is a branched-chain keto acid and an intermediate metabolite of the branched chain amino acid, leucine. Branched-chain amino acids have recently been discovered to be biomarkers of diabetes risk [11]. In the present study, an association between α-ketooctanoic acid levels and IGT was observed. β-ketooctanoic acid is another fatty acid that is formed from the precursor molecule, malonyl-CoA, and may be another metabolite related to identified κeto acid. Higher levels of β-ketooctanoic acid have been detected in subjects with obesity and diabetes [33], [34]. Therefore, it is possible that at least one, or both, of these metabolites contributed to the _mb_GTT performed.

Another metabolite identified in the present study based on its high AUC value was p-cresol sulfate, which is a microbial metabolite that likely derives from secondary metabolism of p-cresol. Diabetic patients have previously been shown to have higher concentrations of both free and total p-cresol concentrations in their blood [35].

Ornithine, another metabolite associated with IGT, is an amino acid produced by the urea cycle with the release of urea from arginine. It was previously established that plasma ornithine concentrations are higher in diabetic subjects [36], and this is a marker of arginase activity. In general, the latter tends to be lower in diabetic patients [36].

Phosphoglycolic acid is a substrate for triose-phosphate isomerase, and an increase in its levels has not previously been associated with IGT.

Butanediol is produced by a variety of microorganisms during a process known as butanediol fermentation [37]. This process involves the anaerobic fermentation of glucose and butanediol is one of the end products. It is possible that this metabolite is reflected in the IGT pattern since gut microbiota have been shown to play a role in the development of type 2 diabetes [38].

Of the metabolites of IGT that exhibited lower levels, changes in potassium levels were a key finding. Previously it was shown that potassium loss occurs with diabetic ketoacidosis. Specifically, there is an obligate loss of positively charged potassium ions from kidney tubules due to increased levels of negatively charged ketones present during IGT. The results of the present study are consistent with these findings.

Although the levels of other metabolites were also observed to decrease, the identification of these metabolites was not successful. ESI is a technique that can include the addition of H^+^, K^+^, and Na^+^ to substances being subjected to mass spectrometry. In the present study, H^+^ and Na^+^ levels were found to be constant in all samples. In contrast, K^+^ (Table 2), and as a consequence all potassium-containing quasi-ions, exhibited high AUC values for the IGT samples. Therefore, additional studies using a technique other than ESI-DIMS are needed to identify the other metabolites associated with IGT that undergo a decrease in blood plasma levels.

It should be noted, that metabolic pattern of IGT, intended for diagnostics by direct infusion mass spectrometry, and metabolite identification results (Table 2) should not exactly match each other. There is not capacity to identify all metabolites in IGT pattern. Especially it concerns low-abundant metabolites which may have high diagnostic power but do not show clear isotopic pattern in mass spectrum or metabolite interference disturbs isotopic pattern in mass spectrum. Moreover, metabolite interference may lead to situation when not first metabolite's isotope (i.e. the reference molecular weight of metabolite in database) is included in IGT pattern.

## Conclusions

In conclusion, the metabolites identified by ESI-DIMS of blood plasma samples from IGT patients and healthy controls confirm that the _mb_GTT is affected by the metabolite signature of blood plasma, and can indicate the development of diabetes in its early stages. Additional validation of this _mb_GTT are needed using a larger population. However, it is anticipated that the _mb_GTT may replace the OGTT for establishing a diagnosis of IGT based on the improved reproducibility of this test that is also more rapid to conduct and more patient friendly.

Future studies are also needed to validate the potential for capillary blood and its dried droplets to be used for this test. The small volume of sample that is required for direct infusion mass spectrometry suggests that capillary blood could be used to perform the _mb_GTT. Currently, dried droplets of blood are generally accepted for metabolic blood analyses. Therefore, if there are difficulties in performing the _mb_GTT in clinical laboratories, the use of dried capillary blood could be an alternative means by which samples could be transported to centralized laboratories that would perform the _mb_GTT.

## Supporting Information

Table S1 **Clinical characteristics of patients.**
(DOC)

Supporting Information S1 **Mass spectra of blood plasma metabolites obtained by direct infusion mass spectrometry (DIMS) of blood plasma samples from control subjects (n = 30) and patients with impaired glucose tolerance (n = 20).**
(ZIP)
