# Supplementary material for: Diagnosing Impaired Glucose Tolerance Using Direct Infusion Mass Spectrometry of Blood Plasma
Source: PLoS One. 2014 Sep 9;9(9):e105343. doi: 10.1371/journal.pone.0105343 (PMC4159123; doi:10.1371/journal.pone.0105343)
Supplement: Table S1 — Clinical characteristics of patients. (DOC) [file pone.0105343.s001.doc]

**Clinical characteristics of patients**

| **Sample #** | **Mass spectrum file name** | **Age**  **(years)** | **HbA1c**  **(%)** | **BMI**  **(kg/m^2^)** | **Cholesterol**  **(mmol/l)** | **LDL**  **(mmol/l)** | **HDL**  **(mmol/l)** | **Triglycerides**  **(mmol/l)** | **Uric acid**  **(µmol/l)** | **Fasting glucose**  **(mmol/l)** | **Insulin**  **(µU/ml)** | **Glucose in OGTT (mmol/l)** | **Sex** | **Status** |
| --- | --- | --- | --- | --- | --- | --- | --- | --- | --- | --- | --- | --- | --- | --- |
| 1 | Sample 1.xml | 66 | 6.3 | 26.91 | 6.7 | 4.7 | 1.2 | 1.7 | 359.5 | 5.5 | 5.5 | 10.5 | male | IGT |
| 2 | Sample 2.xml | 69 | 5.4 | 32 | 5.03 | 3.41 | 1.54 | 1.23 | 351.8 | 5 | 6.1 | 8.2 | female | IGT |
| 3 | Sample 3.xml | 46 | 6.6 | 34.72 | 6 | 3.4 | 1 | 3.4 | 317.4 | 5.8 | 25.8 | 11 | male | IGT |
| 4 | Sample 4.xml | 66 | 5.6 | 36.16 | 4.3 | 2.1 | 1.3 | 2.1 | 581.7 | 5.4 | 25.1 | 10.3 | male | IGT |
| 5 | Sample 5.xml | 55 | 6.2 | 33 | 6.8 | 4.3 | 1.9 | 0.9 | 291.2 | 5.2 | 4.9 | 9.8 | female | IGT |
| 6 | Sample 6.xml | 74 | 6.2 | 27.7 | 3.6 | 1.7 | 1.5 | 0.8 | 369.8 | 5.5 | 9.1 | 8.9 | male | IGT |
| 7 | Sample 7.xml | 73 | 5.7 | 27.95 | 5.3 | 3.2 | 1.4 | 1.5 | 345.3 | 5.9 | 13.6 | 9.2 | female | IGT |
| 8 | Sample 8.xml | 85 | 6.3 | 23.15 | 3.5 | 2.1 | 1.04 | 0.9 | 283.9 | 6.1 | 11.2 | 8.2 | male | IGT |
| 9 | Sample 9.xml | 56 | 6.2 | 24.5 | 3 | 1.9 | 0.67 | 0.9 | 381.3 | 5.4 | 5.8 | 9.8 | male | IGT |
| 10 | Sample 10.xml | 71 | 6.3 | 24.39 | 4.1 | 2.6 | 1.18 | 0.8 | 318 | 5.5 | 7.2 | 10.2 | female | IGT |
| 11 | Sample 11.xml | 55 | 6.4 | 57.01 | 4.5 | 3.1 | 0.8 | 1.3 | 473 | 5.02 | 21.56 | 8.9 | male | IGT |
| 12 | Sample 12.xml | 67 | 6.1 | 25.8 | 6.2 | 4 | 0.9 | 2.9 | 400 | 6.1 | 20.5 | 11 | female | IGT |
| 13 | Sample 13.xml | 38 | 6.4 | 35.89 | 6.7 | 1.6 | 0.55 | 3.9 | 477.8 | 5.8 | 12.4 | 9.2 | male | IGT |
| 14 | Sample 14.xml | 60 | 5.6 | 36.8 | 4 | 2.4 | 0.73 | 1.9 | 452.8 | 5.8 | 22.2 | 10.9 | female | IGT |
| 15 | Sample 15.xml | 81 | 6 | 38.9 | 4.5 | 2.8 | 1.36 | 0.8 | 265.6 | 5.6 | 5.8 | 11 | female | IGT |
| 16 | Sample 16.xml | 53 | 6.6 | 42.18 | 6 | 4.3 | 1.14 | 1.3 | 297.9 | 5.5 | 15.6 | 9.8 | female | IGT |
| 17 | Sample 17.xml | 45 | 6.2 | 50.17 | 6.13 | 4.48 | 0.85 | 1.78 | 402 | 6.1 | 31.7 | 8.4 | female | IGT |
| 18 | Sample 18.xml | 66 | 6.5 | 31.15 | 3.8 | 2.1 | 0.77 | 2.1 | 463 | 5.2 | 14.3 | 11 | male | IGT |
| 19 | Sample 19.xml | 54 | 6.5 | 41.3 | 4.88 | 3.35 | 0.96 | 1.26 | 478.1 | 5.39 | 19.2 | 9.8 | female | IGT |
| 20 | Sample 20.xml | 55 | 5.7 | 28.4 | 6 | 4.3 | 0.6 | 2.4 | 421.2 | 6 | 13.5 | 9 | male | IGT |
| 21 | Sample 21.xml | 61 | 5.5 | 30.61 | 4.4 | 2.8 | 1.4 | 0.5 | 282.8 | 5.9 | 8.1 | 5.1 | male | norma |
| 22 | Sample 22.xml | 48 | 5.4 | 35.51 | 4.6 | 2.7 | 0.8 | 1.5 | 456 | 5.1 | 3.7 | 6.4 | male | norma |
| 23 | Sample 23.xml | 34 | 5.5 | 27.45 | 4.04 | 2.22 | 1.49 | 0.72 | 346.4 | 5.8 | 7.5 | 7.4 | male | norma |
| 24 | Sample 24.xml | 56 | 6.1 | 24.97 | 3.9 | 1.7 | 1.9 | 0.7 | 270.1 | 5.6 | 3.3 | 5.5 | female | norma |
| 25 | Sample 25.xml | 63 | 6.4 | 25.31 | 5.3 | 3.1 | 0.9 | 2.7 | 339.7 | 5 | 13.2 | 6.5 | male | norma |
| 26 | Sample 26.xml | 43 | 5.7 | 46.54 | 5.4 | 3.6 | 1.4 | 1 | 317.4 | 4.8 | 4.8 | 7.2 | female | norma |
| 27 | Sample 27.xml | 56 | 5.1 | 49.32 | 4.7 | 3.3 | 0.74 | 1.7 | 514.3 | 5.4 | 21 | 7.8 | male | norma |
| 28 | Sample 28.xml | 82 | 6.3 | 34.18 | 5.2 | 3.4 | 1.16 | 1.4 | 291.4 | 5.6 | 20.1 | 7.8 | female | norma |
| 29 | Sample 29.xml | 42 | 6.3 | 48.1 | 6.6 | 5.3 | 0.73 | 1.4 | 455 | 5.6 | 17.1 | 6.8 | male | norma |
| 30 | Sample 30.xml | 33 | 5.4 | 38.06 | 5.9 | 4.1 | 1.21 | 1.4 | 309 | 5.8 | 17.3 | 5.2 | female | norma |
| 31 | Sample 31.xml | 75 | 6 | 33.91 | 4.5 | 3 | 0.84 | 1.5 | 446.4 | 5.4 | 12.9 | 6.8 | female | norma |
| 32 | Sample 32.xml | 43 | 5.5 | 53.2 | 4.7 | 2.7 | 1.23 | 1.6 | 425.5 | 5.1 | 20.2 | 7.1 | female | norma |
| 33 | Sample 33.xml | 37 | 6 | 30.1 | 4.8 | 3 | 1.27 | 1.1 | 309 | 6.1 | 7.8 | 6.5 | female | norma |
| 34 | Sample 34.xml | 60 | 6.1 | 41.6 | 4.7 | 3.03 | 0.85 | 1.28 | 379 | 5.8 | 18.5 | 6.5 | male | norma |
| 35 | Sample 35.xml | 65 | 6 | 37.46 | 5.8 | 4.1 | 0.98 | 1.6 | 491 | 5.5 | 15.5 | 5.4 | female | norma |
| 36 | Sample 36.xml | 36 | 5.8 | 45.5 | 5.06 | 3.56 | 0.69 | 1.8 | 459.2 | 5.3 | 29.9 | 5.8 | male | norma |
| 37 | Sample 37.xml | 32 | 5.1 | 41.55 | 4.4 | 3 | 0.91 | 1.1 | 375.4 | 5.4 | 100.8 | 6.8 | male | norma |
| 38 | Sample 38.xml | 62 | 5.8 | 34.8 | 5.9 | 4 | 1.4 | 1.2 | 498.6 | 5.5 | 15.2 | 6.1 | female | norma |
| 39 | Sample 391.xml | 35 | 5.5 | 40 | 5.6 | 3.8 | 1 | 1.7 | 443.8 | 5.7 | 62.5 | 7.7 | female | norma |
| 40 | Sample 40.xml | 50 | 6 | 41.43 | 6.4 | 4.2 | 0.8 | 3 | 439.2 | 6.1 | 15 | 7.3 | male | Norma |
| 41 | Sample 41.xml | 53 | 5.8 | 35 | 5.2 | 3.4 | 1.2 | 1.3 | 374 | 6.4 | 16.1 | 6.4 | female | Norma |
| 42 | Sample 42.xml | 43 | 5.9 | 25.94 | 5.2 | 3.4 | 1.4 | 0.9 | 223.3 | 5.7 | 7.2 | 5.7 | female | norma |
| 43 | Sample 43.xml | 39 | 5.3 | 25.55 | 5.9 | 3.8 | 1.8 | 0.6 | 261 | 5.1 | 4.2 | 5.8 | female | norma |
| 44 | Sample 44.xml | 55 | 5.7 | 26.36 | 5.9 | 4.3 | 1.3 | 0.7 | 329.8 | 5.2 | 3.6 | 5.9 | female | norma |
| 45 | Sample 45.xml | 70 | 6.3 | 29.74 | 5.7 | 4 | 1 | 1.5 | 353 | 5.7 | 7.2 | 7.3 | male | norma |
| 46 | Sample 46.xml | 58 | 5.8 | 31.6 | 6.2 | 4.2 | 1.6 | 0.9 | 235.7 | 5 | 4.3 | 7.5 | female | norma |
| 47 | Sample 47.xml | 66 | 5.6 | 38.72 | 3.9 | 3.41 | 1.13 | 1.3 | 459.8 | 5.6 | 11.4 | 4.8 | male | norma |
| 48 | Sample 48.xml | 59 | 5.8 | 26.89 | 5.92 | 3.88 | 1.69 | 0.6 | 422 | 5.4 | 4.5 | 4.1 | male | norma |
| 49 | Sample 49.xml | 67 | 5.9 | 26.02 | 4.1 | 2.4 | 1.1 | 1.4 | 376.5 | 5.5 | 5.7 | 7.7 | male | norma |
| 50 | Sample 50.xml | 75 | 5.3 | 24.55 | 6.3 | 3.9 | 1.9 | 1.2 | 345.7 | 5.6 | 3.4 | 5.7 | male | norma |
